# Complementary molecular methods reveal comprehensive phylogenetic diversity integrating inconspicuous lineages of early-diverged wood-decaying mushrooms

**DOI:** 10.1038/s41598-020-59620-0

**Published:** 2020-02-20

**Authors:** Takashi Shirouzu, Shunsuke Matsuoka, Hideyuki Doi, Nobuaki Nagata, Masayuki Ushio, Kentaro Hosaka

**Affiliations:** 1 Graduate School of Bioresources, Mie University, 1577 Kurima-machiya, Tsu, Mie 514-8507 Japan; 2 Graduate School of Simulation Studies, University of Hyogo, 7-1-28 Minatojima-minamimachi, Chuo-ku, Kobe, Hyogo 650-0047 Japan; 3 Collection Center, National Museum of Nature and Science, 4-1-1 Amakubo, Ibaraki, 305-0005 Japan; 4 Hakubi Center, Kyoto University, Kyoto, 606-8501 Japan; 5 Center for Ecological Research, Kyoto University, Hirano 2-509-3, Otsu, Shiga 520-2113 Japan; 6 PRESTO, Japan Science and Technology Agency, Kawaguchi, 332-0012 Japan; 7 Department of Botany, National Museum of Nature and Science, 4-1-1 Amakubo, Tsukuba, Ibaraki 305-0005 Japan

**Keywords:** Microbial ecology, Fungal ecology

## Abstract

Among terrestrial microorganisms, mushroom-forming fungi have been relatively well investigated, however the inconspicuous strains may be overlooked by conventional visual investigations causing underestimation of their phylogenetic diversity. Herein, we sought to obtain a comprehensive phylogenetic diversity profile for the early-diverging wood-decaying mushrooms Dacrymycetes, using an approach that combines fruiting-body collection, culture isolation, and environmental DNA (eDNA) metabarcoding of decaying branches. Among the 28 operational taxonomic units (OTUs) detected during a three-year investigation, 10 each were from fruiting bodies and cultured mycelia and 27 were detected as eDNA sequences. eDNA metabarcoding revealed various lineages across the Dacrymycetes phylogeny. Alternatively, fruiting-body and culture surveys uncovered only ~50% of the OTUs detected through eDNA metabarcoding, suggesting that several inconspicuous or difficult-to-isolate strains are latent in the environment. Further, eDNA and culture surveys revealed early-diverging clades that were not identified in the fruiting-body survey. Thus, eDNA and culture-based techniques can uncover inconspicuous yet phylogenetically important mushroom lineages that may otherwise be overlooked via typical visual investigations.

## Introduction

Microorganism diversity has been challenging to comprehensively investigate owing to their microscopic-size and high species diversity. This statement generally applies to many terrestrial microorganisms. However, mushroom-forming fungi are an exception as they grow visible fruiting bodies, allowing for their diversity and phylogeny to be relatively well studied^1^. Mushroom diversity has been investigated using morphological and phylogenetic analyses based on collected fruiting bodies. However, in most mushroom species, fruiting bodies appear in a temporally sporadic manner, with the small and ephemeral fruiting bodies frequently being overlooked. Therefore, despite the importance of mushroom-forming fungi as decomposers or symbionts in terrestrial ecosystems, a large proportion of the mushroom species diversity remains poorly described^2^. Furthermore, the early-diverged lineages of mushroom-forming fungi tend to feature inconspicuously small or thin fruiting bodies^1,3,4^. Thus, even though the fruiting bodies may not always be detectable within a sampling site, various mushroom strains may nevertheless inhabit the same location as inconspicuous mycelia^4,5^. These lineages account for an underestimation of species diversity, thereby preventing the reconstruction of a comprehensive phylogeny. Consequently, for an accurate and comprehensive description of mushroom-forming fungi diversity, the inconspicuous mycelia of these fungi must be investigated in addition to their visible fruiting bodies.

Two methods are generally employed for detecting fungal mycelia from environmental samples: culture isolation^6,7^ and environmental DNA (eDNA) analysis^8–11^. The former method allows for detection of fungal diversity without the collection and identification of visible fruiting bodies and concurrently enables the collection of culturable mycelia. However, several strains are not readily isolated using this method as culture conditions are often inappropriate for their growth^12,13^. Alternatively, fungal eDNA is defined as the DNA derived from fungal cells present in environmental samples such as soil and decaying wood. A notable contribution from fungal eDNA surveys is the discovery of evolutionarily significant lineages, including early-diverged fungi such as Cryptomycota^14–16^ and Archaeorhizomycetes^17,18^, which are otherwise challenging to detect using traditional methods. The eDNA metabarcoding method, which employs high-throughput sequencing (HTS), typically generates millions to billions of DNA sequence reads and efficiently detects fungal DNA in environmental samples. The high-throughput nature of this method and the recent reduction in sequencing costs has allowed eDNA metabarcoding to emerge as a widely used and powerful technique in the surveying of fungal diversity^19,20^.

The various methods designed for the detection of fruiting bodies, isolated mycelia, and eDNA provide complementary biological information regarding fungal taxonomic and phylogenetic diversity and ecological features. The diversity of mushroom-forming fungi has previously been investigated using methods based on the fruiting-body, culture isolation, and/or eDNA^4,21–27^. These studies have revealed the unique characteristics of each method. For example, the detected fungal community compositions (detected as operational taxonomic units; OTUs) differ between methods, with eDNA-based methods tending to record OTU richness higher than those from the other methods. However, few studies have compared both taxonomic and phylogenetic diversities by estimating the phylogenetic positions of the OTUs obtained from simultaneous investigations using each method.

As a first step in identifying a favourable research framework for surveying fungal diversity, the detection performance of distinct methods must be concurrently examined. Herein, to compare the phylogenetic diversities detected using different methods, we have focused on Dacrymycetes (Agaricomycotina, Basidiomycota), as this taxon is a monophyletic group that contains approximately 120 species, which is a manageable study size. Moreover, the taxon is a noteworthy lineage for studying the evolution of wood-decaying mushrooms. Dacrymycetes species are considered to represent an ancestral lineage of wood-decaying basidiomycetes that diverged approximately 350 million years ago^1,28,29^ and brown rot fungi that are critical for understanding the early evolution of fungal wood decomposers^30^. Diversity surveys and phylogenetic analyses of Dacrymycetes have been conducted primarily based on visible fruiting bodies^29–32^. Thus, the phylogenetic position of newly obtained dacrymycetous sequences can be estimated based on accumulating reference sequences. However, species belonging to the early branches in the Dacrymycetes phylogeny tend to grow inconspicuously small or thin fruiting bodies^4^, which suggests the existence of unknown lineages that cannot be readily detected through visual investigations.

In a previous study, we investigated the diversity of Dacrymycetes over one year using three methods: fruiting-body collection, culture isolation followed by DNA sequencing, and eDNA analysis of decaying branches through DNA cloning^4^. eDNA-cloning and culture isolation methods detected OTUs belonging to unknown clades in the phylogeny that were not identified among the sequences obtained via the fruiting-body survey. However, certain OTUs were detected only as fruiting bodies^4^. As the study period was short (one year) and the sample size was small, the number of OTUs detected was not saturated. Moreover, some lineages could not be detected probably owing to primer mismatches in the previous study^4^, and the sequence reads generated through DNA cloning were limited. Therefore, it has remained unclear whether our previous findings are caused by differences in methods or insufficient sampling. Although the occurrence of wood-decaying fungi might be affected by environmental factors such as investigation site, seasonality, and decomposition stage of substratum^7–9^, the previous study only investigated one site for one year, and the environmental effect on the diversity surveyed by each method was not fully examined.

To more accurately and robustly evaluate fungal phylogenetic diversity, our aim in this study was to survey fungal diversity using a combined and improved approach. We investigated Dacrymycetes for three years by examining the performance of three distinct methods, i.e. fruiting-body collection, culture isolation using a dilution-to-extinction method, and amplicon-based eDNA metabarcoding with degenerate primers and HTS. To assess OTU diversity and phylogenetic characteristics obtained using each method, the detected taxonomic and phylogenetic diversities were compared among the three methods. In addition, we evaluated the effects of environmental variables (forest age, decomposition stage, season, and year), which are thought to affect the diversity detected by each method. Based on the obtained results, we discuss the advantages and disadvantages of each method for efficiently surveying fungal diversity.

## Results

### Obtained samples, DNA sequences, and OTUs

We collected 75 fruiting bodies and isolated 49 cultures of Dacrymycetes via fruiting-body collection and culture isolation using a dilution-to-extinction method over the course of a three-year investigation in a *Pinus densiflora* forest. MiSeq sequencing of the eDNA library constructed from *P. densiflora* decayed-branch samples using a primer designed for large subunit (LSU) ribosomal DNA (rDNA) of Dacrymycetes generated 7,039,142 reads, of which 6,918,978 were retained after quality filtering and denoising. The filtered reads were grouped into 1,679 OTUs. After sequence read counts with nine reads or less were eliminated, 6,776,985 reads belonging to 915 OTUs remained, and subsequent exclusion of OTUs detected in <4 replicates, resulted in 6,601,824 final reads in 493 OTUs. Among these remaining OTUs, 32 were identified as members of Dacrymycetes using BLAST. Although the UCHIME algorithm did not identify these OTUs as chimeric sequences, we considered six of the 32 OTUs as chimeric owing to anomalous long branches observed during preliminary phylogenetic analyses. Additionally, the first- and second-half sequences were distantly related at the class level. eDNA sequences from our previous study^4^ were re-examined based on the same criteria, and LC003976, LC003977, and LC003993 (Clades L, M, and J, respectively) were determined to be chimeric sequences. Thus, we prevented further public disclosure of these sequences within the DNA Data Bank of Japan (DDBJ). OTU_266 was found to exhibit high sequence similarity to *Ceratobasidium pseudocornigerum* (MG002432), which is a species of Agaricomycetes. However, we concluded that this OTU should be a member of Dacrymycetes as it was included in the dacrymycetous clade (Supplementary Fig. 1).

**Figure 1:**
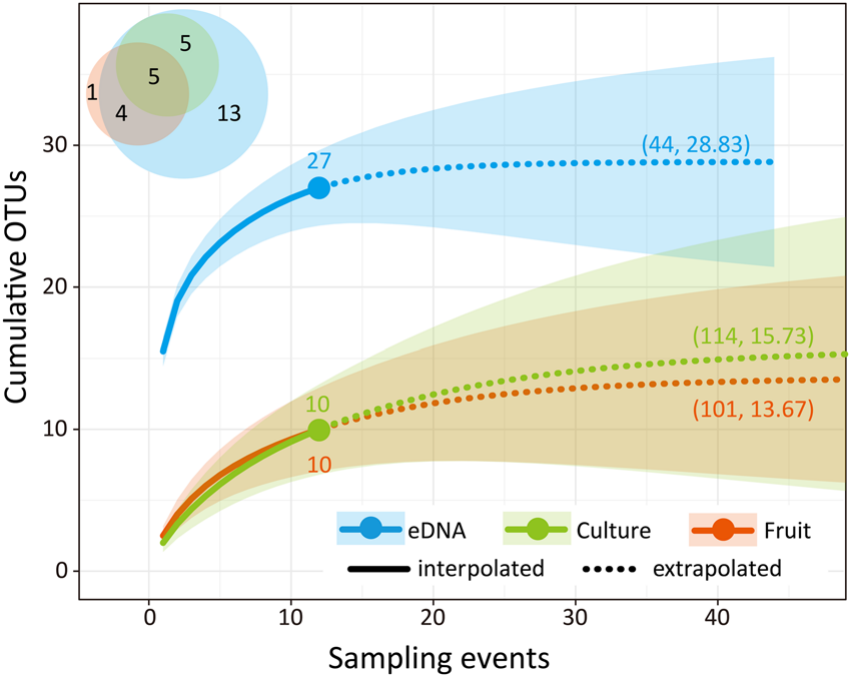
Rarefaction curves with 95% confidence intervals (shaded areas, based on a bootstrap method applied with 100 replications) and Venn diagram based on number of OTUs detected as fruiting bodies (as “Fruit” in the figure), cultures, and eDNA sequences. Numbers in parentheses: the estimated sampling event when the OTU number was saturated and the estimated OTU number.

After careful examination of all the OTUs detected using the three methods, 28 OTUs were accepted as Dacrymycetes. Among these, 10 were collected as fruiting bodies, 10 were isolated as cultured mycelia, and 27 were detected as eDNA sequences (Fig. 1). Further, eDNA metabarcoding identified nine of the 10 OTUs collected as fruiting bodies and all 10 OTUs isolated as cultured mycelia. Conversely, 13 OTUs were detected only based on eDNA analysis, while one OTU was collected only through the fruiting-body survey. The generated rarefaction curves showed that the cumulative number of OTUs saturated at 28.83, 15.73, and 13.67 for the eDNA analysis, culture isolation, and fruiting-body collection, respectively (Fig. 1). The three methods were expected to detect the following numbers of OTUs in four sampling events (May, July, September, and November in one year) and 12 sampling events (over three years): eDNA analysis, 22.13 OTUs (77% of 28.83) and 27 OTUs (94% of 28.83), respectively; fruiting-body collection, 6.02 OTUs (44% of 13.67) and 10 OTUs (73% of 13.67), respectively, and culture isolation, 5.31 OTUs (34% of 15.73) and 10 OTUs (64% of 15.73), respectively.

### Molecular phylogeny

**Figure 2:**
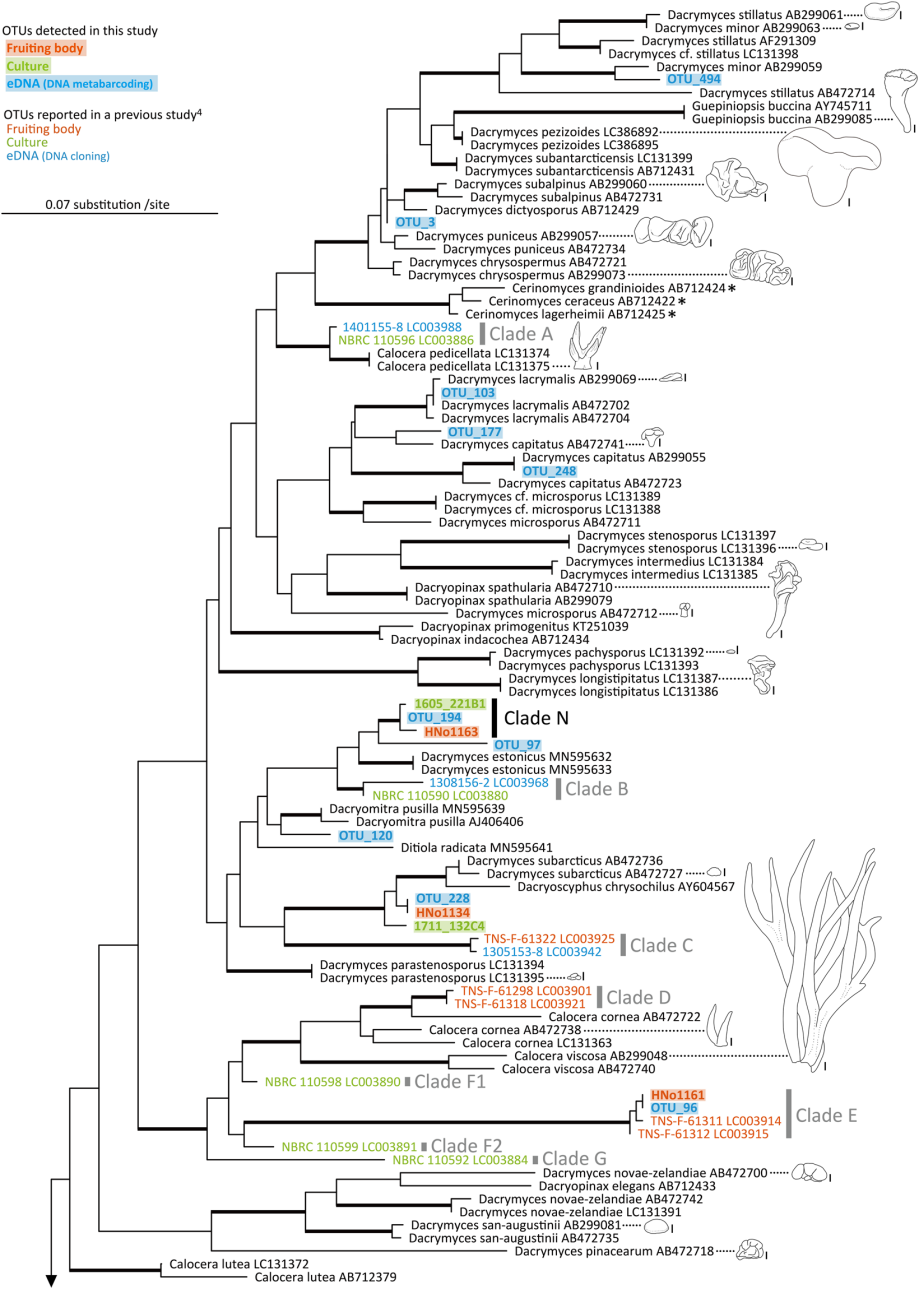
Phylogenetic tree of Dacrymycetes estimated by using RAxML with previously known and newly obtained large subunit ribosomal DNA (LSU rDNA) sequences. Thick branches indicate maximum-likelihood bootstrap percentages (MLBP) ≥ 80%. Clades N is a lineage newly detected in this study. Clades A–G were reported in a previous study^4^. Line drawings: fruiting bodies of Dacrymycetes; scale bars in line drawings: 1 mm. *, inconspicuous species featuring thin resupinate fruiting bodies.

**Figure 3:**
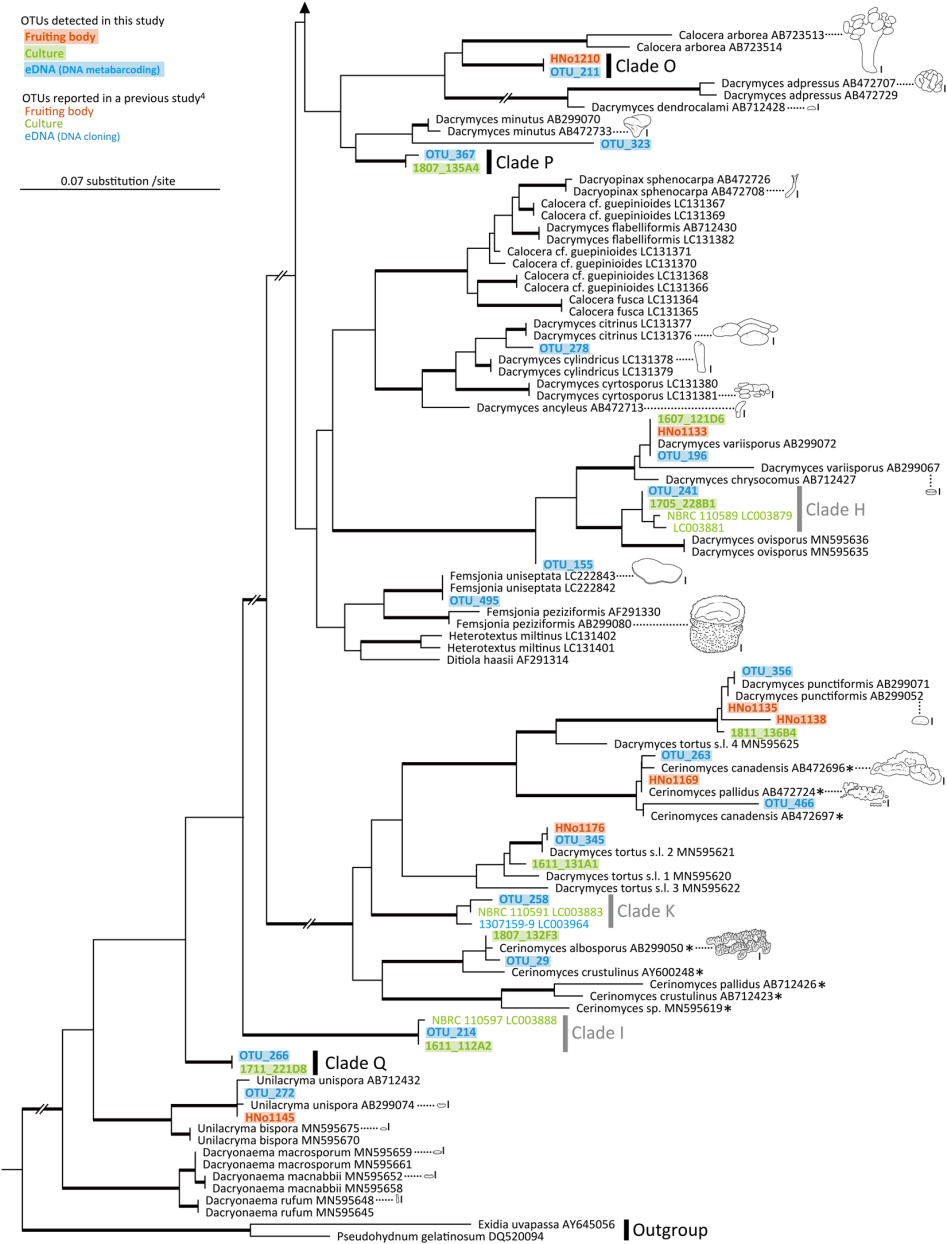
Continued from Fig. 2. Phylogenetic tree of Dacrymycetes estimated by using RAxML with previously known and newly obtained large subunit ribosomal DNA (LSU rDNA) sequences. Thick branches indicate maximum-likelihood bootstrap percentages (MLBP) ≥80%. Clades O–Q are lineages newly detected in this study. Clades H, I, and K were reported in a previous study^4^. Line drawings: fruiting bodies of Dacrymycetes; scale bars in line drawings: 1 mm. *, inconspicuous species featuring thin resupinate fruiting bodies. A phylogenetic tree with unedited branch lengths is shown in Supplementary Fig. 1.

The eDNA metabarcoding method detected diverse lineages throughout the Dacrymycetes phylogeny, including lineages closely related to all OTUs collected as fruiting bodies or isolated mycelia (Figs. 2, 3 and Supplementary Fig. 1). The newly detected clades in this study were clade N (HNo1163, 1605_221B1, and OTU_194), O (OTU_211 and HNo1210), P (OTU_367 and 1807_135A4), and Q (OTU_266 and 1711_221D8). Conversely, OTU_96 and HNo1161, OTU_241 and 1705_228B1, OTU_214 and 1611_112A2, and OTU_258 formed monophyletic groups with previously reported clades E, H, I, and K, respectively^4^. Furthermore, OTU_494, _103, _248, and _495 formed monophyletic groups with their sequences identified as *Dacrymyces minor*, *Dacrymyces lacrymalis*, *Dacrymyces capitatus*, and *Femsjonia uniseptata*, respectively; OTU_356, HNo1135, HNo1138, and 1811_136B4 formed a monophyletic group with their sequences identified as *Dacrymyces punctiformis*; OTU_345 and HNo1176 formed a monophyletic group with their sequences identified as *Dacrymyces tortus* s.l. 2; and OTU_272 and HNo1145 formed a monophyletic group with their sequences identified as *Unilacryma unispora*.

### Factors affecting richness and composition of detected OTUs

**Figure 4:**
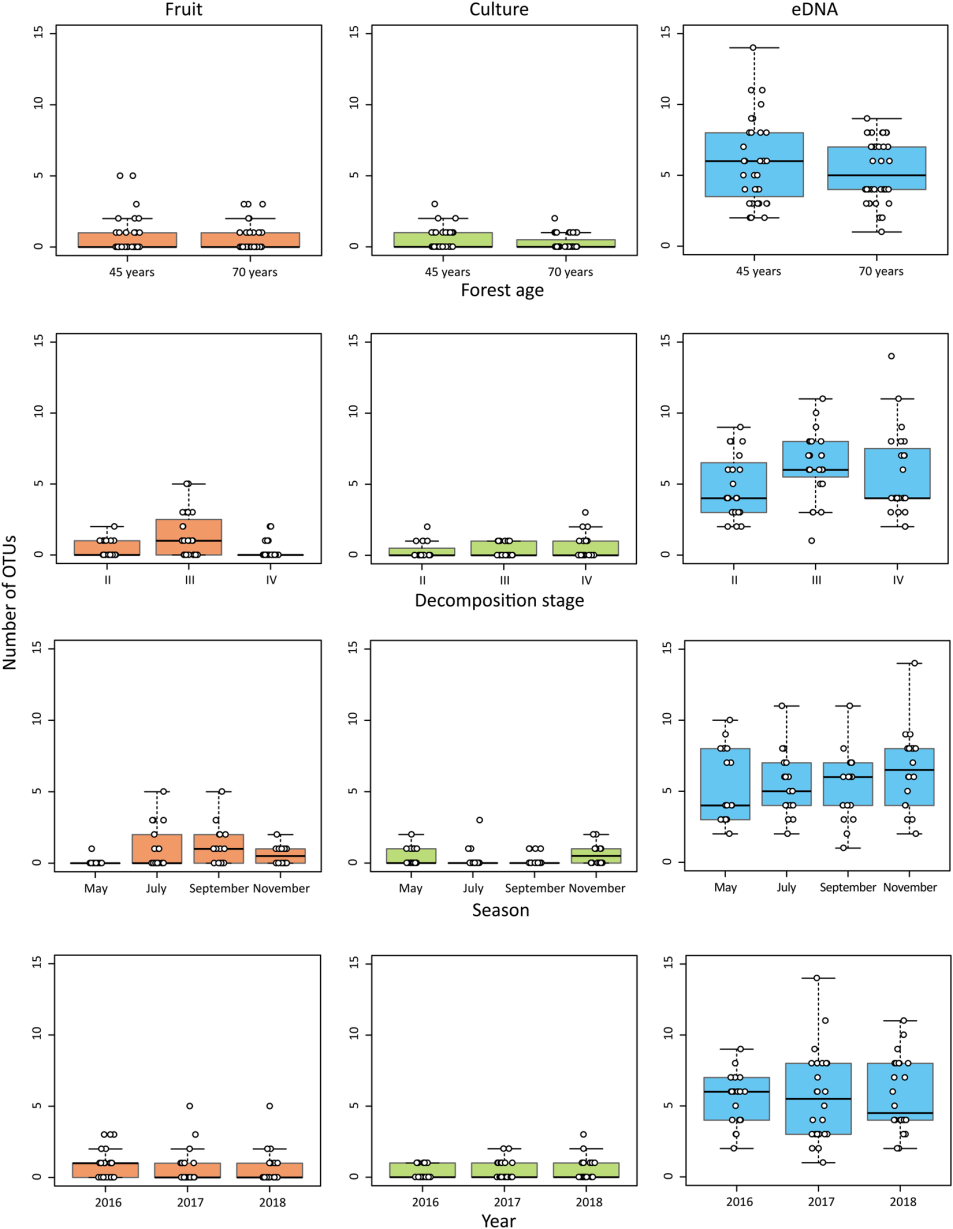
Box plots of detected OTUs disaggregated by forest age, decomposition stage, season, and year. Fruiting-body is shown as “Fruit” in the figure. The top border of the box indicates the 25^th^ percentile, the black line within the box marks the median, and the bottom end of the box indicates the 75^th^ percentile. Whiskers above and below the box indicate the largest value within 1.5 times the interquartile range above the 75^th^ percentile and the smallest value within 1.5 times the interquartile range below the 25^th^ percentile. White circles are individual data points.

The detection method (fruiting-body collection, culture isolation, or eDNA analysis), forest age of plots (45 or 70 years), decomposition stage of branches (II, solid; III, decayed, and IV, fragile), sampling season (May, July, September, or November), and year (2016, 2017, or 2018), which could affect OTU richness and composition, were chosen as explanatory variables, and their effects on OTU richness and composition were examined based on the generalised linear model (GLM) and permutational multivariate analysis of variance (PERMANOVA), respectively. To evaluate phylogenetic diversity, standard effect sizes (SESs) of the mean pairwise distance (MPD) and the mean nearest taxon distance (MNTD) were calculated. The number of detected OTUs was sorted by forest age, decomposition stage, season, and year (Fig. 4). GLM results showed that the method alone significantly affected OTU richness in the complete dataset (Table 1, *P* < 0.001). Further, Tukey’s test revealed significant differences in all combinations of the methods (Table 2, α < 0.05), and OTU richness was the highest in the case of eDNA metabarcoding, followed by those in fruiting-body collection and culture isolation. The results of Akaike information criteria corrected for small sample size (AICc)-based model selection showed that distinct variables were selected among the survey methods. Seasons were selected for the fruiting-body collection, with OTU richness being high in July and September and low in May and November (Table 3, Supplementary Tables 2–6); forest age was selected for the culture isolation method, with OTU richness being low in the older forest; no environmental factor was selected for the OTU richness detected using eDNA analysis. Decomposition stage and year were selected for MPD (SES) and MNTD (SES) of the eDNA analysis, and these phylogenetic diversity metrics decreased with substratum decomposition and varied from year to year. PERMANOVA results showed that the method (Raup-Crick, *P* = 0.0002; Unifrac, *P* = 0.0004) and decomposition stage (*P* = 0.0002) significantly affected OTU composition in all datasets (Table 4). In the case of the Raup-Crick metric, decomposition stage was determined to have a significant effect for fruiting-body collection (*P* = 0.0184), while Raup-Crick and Unifrac metrics revealed that decomposition stage had a significant effect for the eDNA analysis (Raup-Crick, *P* = 0.0002; Unifrac, *P* = 0.0008).

**Table 1:** Results of GLM examining the effects of methods and environmental factors on detected OTU richness.

	Df	Deviance	Resid. Df	Resid. Dev	Pr (>Chi)
NULL			215	789.90	
Method	2	529.13	213	260.77	<2e-16***
Forest age	1	1.76	212	259.02	0.1850
Decomposition stage	1	1.66	211	257.36	0.1980
Season	3	5.43	208	251.93	0.1428
Year	2	0.05	206	251.87	0.9733

***P < 0.001.

Df, degrees of freedom; Resid., residual; Dev, deviance.

**Table 2:** Results of Tukey’s test of GLM for detection of OTU richness differences between methods. Fruiting-body is shown as “Fruit” in the table.

	Estimate	Std. Error	z value	Pr (>|z|)
eDNA × Culture	2.6642	0.1955	13.631	<1e-04***
Fruit × Culture	0.5596	0.2369	2.362	0.0453*
Fruit × eDNA	−2.1046	0.1513	−13.909	<1e-04***

***P < 0.001, *P < 0.05.

**Table 3:** Optimal GLM for the effects of environmental factors on OTU richness, MPD (SES), and MNTD (SES) based on AICc. Fruiting-body is shown as “Fruit” in the table.

		Coefficients									AICc
		Forest age	Decomposition stage	Season				Year			
				May	July	September	November	2016	2017	2018	
Fruit	OTU richness	—	—	−2.9440	0.0541	−4.95E-16	−0.6419	—	—	—	159.8
Culture	OTU richness	−0.0235	—	—	—	—	—	—	—	—	118.3
eDNA	OTU richness	—	—	—	—	—	—	—	—	—	327.8
	MPD (SES)	—	−0.1814	—	—	—	—	0.6547	−0.1237	0.3436	169.4
	MNTD (SES)	—	−0.2888	—	—	—	—	0.6024	−0.6223	0.1802	143.2

Hyphen, Not selected.

**Table 4:** Results (Pr value) of PERMANOVA for the effects of methods and environmental factors on detected OTU compositions. Fruiting-body is shown as “Fruit” in the table.

	Raup-Crick				Unifrac			
	All	Fruit	Culture	eDNA	All	Fruit	Culture	eDNA
Method	0.0002***				0.0004***			
Forest age	0.6143	0.4683	—	0.4685	0.3729	0.6809	—	0.6267
Decomposition stage	0.0002***	0.0184*	—	0.0002***	0.0002***	0.0996	—	0.0008***
Season	0.9076	0.2194	—	0.9644	0.2811	0.6115	—	0.5371
Year	0.5173	0.1268	—	0.4789	0.1283	0.8006	—	0.1308

***P < 0.001, *P < 0.05.

Hyphen, Not available.

## Discussion

The eDNA metabarcoding technique revealed a more comprehensive OTU diversity for the various clades in the Dacrymycetes phylogeny than those of the other two traditional methods, i.e. fruiting-body and culture surveys (Figs. 2, 3). Although one OTU (HNo1138) was detected uniquely in fruiting bodies, its sequence was closely related to that of OTUs detected using eDNA metabarcoding and culture isolation. Moreover, the fruiting-body and culture surveys yielded only approximately half the OTUs detected using eDNA metabarcoding (Fig. 1), suggesting that several inconspicuous or difficult-to-isolate strains are latent in the surveyed environment. eDNA and culture surveys revealed early-diverged clades that were not identified in the fruiting-body survey. Thus, the eDNA and culture-based diversity investigations effectively identified inconspicuous yet phylogenetically important mushroom lineages that may otherwise be overlooked using traditional visual investigative techniques.

In addition to the four clades (clade E, H, I, and K) reported by the previous study^4^, eDNA metabarcoding discovered four new clades (clade N, O, P, and Q), and the OTUs close to *D. lacrymalis* and *Dacrymyces variisporus* that were not detected by the previous DNA cloning. Compared to the previous study, the improved detections of this study would be attributed to the longer-term and larger-area survey, reduction of primer mismatches by using the degenerate primers, and/or acquisition of a much larger number of sequence reads by eDNA metabarcoding with HTS.

The fruiting-body collection tended to yield large fruiting bodies (>2 mm in diameter), some of which were identified as known species (e.g. *D. variisporus* and *D. punctiformis*). Although OTUs belonging to clades H, I, K, P, and Q were detected using the eDNA or culture method, their fruiting bodies were not identified at any time during this investigation. Clades I and Q, *Unilacryma* spp., and *Dacryonaema* spp., which branched deep in the phylogenetic tree, are considered to be important lineages for studying the early evolution of Dacrymycetes^29,33^. Lineages that diverged early in the phylogeny of Dacrymycetes tend to feature small or thin fruiting bodies^4,33^. The fruiting bodies of *Unilacryma* spp. and *Dacryonaema* spp. are as small as ≤ 2 mm in diameter, and *Cerinomyces* spp. harbour inconspicuous thin resupinate basidiocarps^29,31,34,35^. Other mushroom-forming basidiomycetes, Agaricomycetes, and Tremellomycetes, which constitute Agaricomycotina with Dacrymycetes, display variously shaped fruiting bodies, such as stipitate agaricoid, coralloid, and corticoid fruiting bodies, yet their early-diverged lineages tend to feature inconspicuous small or thin fruiting bodies^1,3^. These classes contain species that play ecologically significant roles, e.g. decomposers and symbionts in terrestrial ecosystems, and previous visual investigations have likely overlooked these inconspicuous yet phylogenetically important lineages^1,3^. To perform a comprehensive investigation of the phylogenetic diversity of mushroom-forming fungi, future studies must focus on surveying these inconspicuous early branches in addition to the conspicuous lineages. Our results suggest that the use of eDNA metabarcoding may serve to fill the current void in fungal phylogeny by identifying inconspicuous lineages within various environments.

The eDNA metabarcoding method is expected to stably detect higher OTU diversity than that expected from other approaches, even under fluctuating environmental conditions (Fig. 4, Table 3). Season and forest age affected the variation in OTU richness detected using the fruiting-body and culture isolation methods, respectively (Table 3). As the physiological conditions (e.g. intrinsic growth rate) of mushrooms or mycelia may change depending on environmental conditions, the diversity evaluated based on fruiting bodies or isolated cultures would also likely be susceptible to environmental fluctuations. Considering that eDNA metabarcoding revealed a higher OTU richness (relative to other approaches) regardless of season and forest age (Fig. 4), the eDNA metabarcoding method may serve as a consistently superior method in studies aimed at comprehensively describing fungal diversity.

The eDNA metabarcoding and fruiting-body approaches also detected changes in dacrymycetous communities associated with substrate degradation (Table 4). Decomposition stage significantly affected OTU composition in the Raup-Crick and Unifrac metrics, suggesting that the taxonomic and phylogenetic composition of the detected OTUs differed depending on the degree of decay. The OTU composition changes that correlated with decomposition stages may have occurred because of the fungal succession associated with wood decomposition^8,36^. For example, eDNA metabarcoding yielded OTUs that were detected only at specific decomposition stages (OTU_367, _494, and _495 from IV, II, and III, respectively; Supplementary Table 1), which suggests that the eDNA metabarcoding method could be useful to study the relationship between specific fungal lineages and their ecological functions. In the eDNA metabarcoding analysis, phylogenetic diversity indices (i.e. SES values of MPD and MNTD) were found to decrease as decomposition progressed (Table 3). Considering the high OTU detection by eDNA metabarcoding from all decomposition stages (Fig. 4), this approach may successfully reveal the changes in phylogenetic compositions associated with substratum decomposition that cannot be readily identified using other methods. Although the selected model was that in which MPD (SES) and MNTD (SES) varied from year to year (Table 3), it remains unclear whether the observed pattern was caused by any long-term environmental changes.

In this study, eDNA metabarcoding was shown to be an effective method for detecting high phylogenetic diversity of fungi even during a short-term investigation, without wide fluctuations in environmental conditions. Therefore, eDNA metabarcoding may serve as a suitable method for an initial survey to obtain an overview of fungal diversity. The sampling efforts involved in the fruiting-body and culture surveys appear inadequate (Fig. 1). However, even after saturation, the estimated numbers of OTUs detected using these two methods were roughly half of that obtained using eDNA metabarcoding. Fruiting-body and culture surveys may require relatively long-term and high-density investigations over several years to obtain their target biological samples. By collecting fruiting bodies or isolated mycelia, we can gain biological and ecological insights regarding individual fungal species. For example, fruiting-body morphologies serve as criteria for descriptive taxonomy and may represent the spore-dispersal strategy, while the cultured mycelium is useful for testing physiological properties.

Based on our results, we propose an efficient research framework for fungal diversity surveying that combines these methods: (1) short-term surveying using eDNA metabarcoding to obtain an overview of the fungal diversity in a target area, (2) identification of potential microhabitats of undiscovered lineages based on eDNA metabarcoding results, and (3) a highly focused survey to obtain biological samples such as fruiting bodies and isolated cultures for detailed ecological and physiological descriptions of the fungi. By adopting eDNA metabarcoding as an initial survey tool, we can efficiently compile a list of samples or potential microhabitats of targeted lineages. The proposed research framework would enable efficient investigation of previously undiscovered fungal diversity and identification of their biological entities. Given the continuous and rapid advances in sequencing technology (e.g. long-read sequencing by Pacific Biosciences and Oxford Nanopore Technology), a diversity survey that combines eDNA metabarcoding and phylogenetic analyses is likely to emerge soon as a highly effective and adaptable approach for studying diverse fungal groups. A comprehensive approach combining recently developed and traditional methods will likely provide insights that will serve to link the distinct perspectives of the newly revealed diversity based on eDNA analysis and the traditionally recognised diversity under the Linnaean classification system^19,37^. Therefore, this complementary surveying approach should enable integration of the different views for fungal diversity.

## Methods

### Study site and sample collection

The study site was a mixed forest of *P. densiflora*, a coniferous tree, and broad-leaved deciduous trees at an elevation of ~1,300 m in the Sugadaira Research Station (N36°31′17″, E138°20′60″), University of Tsukuba. According to the Japan Meteorological Agency, statistical data for 1981−2010, the average annual temperature of this area is 6.4 °C, the lowest and highest monthly average temperatures are −6.2 °C in January and 19.5 °C in August, respectively, and the snow season is from October to April. In this forest, two 60 × 60 m plots were established, Plot 1 (~45 years old) and Plot 2 (~70 years old), and in each plot, one of the authors (T. Shirouzu) collected the fruiting bodies of Dacrymycetes and the decaying branches (1−5 cm in diameter) of *P. densiflora* four times per year (in May, July, September, and November) from 2016 to 2018. Dacrymycetous fruiting bodies appearing on the decaying branches (1−5 cm in diameter) of *P. densiflora* were surveyed for 1 h in each plot, and all fruiting bodies found were collected. Some of the obtained fruiting bodies were separated from the substrate, soaked in DMSO buffer^38^ containing 100 mM Tris-HCl (pH 8.0) and 0.1 M sodium sulphate (Na_2_SO_3_), and stored at −20 °C until DNA extraction. The remaining fruiting bodies were dried using a food dehydrator (at 55 °C for 24 h) and deposited in the National Museum of Nature and Science (Supplementary Table 1). The obtained decaying branches of *P. densiflora* were divided into three decomposition stages, i.e. solid, decayed, and fragile (II, III, and IV, respectively)^39^, and four branches were collected for each decomposition stage at each plot (24 branches/sampling event). The collected branches were placed in paper bags and brought to the laboratory, air-dried at room temperature (24−26 °C) for two days, and used for culture isolation and DNA extraction.

### Culture isolation from decaying branches

Culture isolation was performed using a dilution-to-extinction method^40^ modified for wood-decaying fungi^4^. Collected branches were debarked and washed in running tap water. A 10-g segment was cut from each branch, converged with samples in the same decomposition stage from the same plot, and pulverised with 500 mL of distilled water in a blender (7011HS, Waring Commercial, Torrington, CT, USA) for 1 min at the high speed setting. The pulverised sample was passed through four sieves (mesh sizes: 500, 300, 212, and 106 µm) with running distilled water by using an electric sieve shaker (M-3T, Tsutsui Scientific Instruments Co., Ltd., Tokyo, Japan), and the particles that aggregated in the 106-µm sieve were collected. Next, a 2-mg sample of the particles was transferred into a 50-mL centrifuge tube, and the remainder was dried and stored with silica gel at −20 °C and used for DNA extraction. For washing particles, 20 mL of distilled water were added to the centrifuge tube and centrifuged at 2,200 × *g* for 3 min in a tabletop centrifuge (Model 4000, KUBOTA, Tokyo, Japan). The supernatant was then removed from the tube and 20 mL of fresh distilled water were added. This washing process was repeated 10 times. The washed particles were diluted with a 1% solution of CMC (carboxymethyl cellulose, No. 1190, Daicel FineChem Ltd., Tokyo, Japan) to a concentration of 1−2 particles/50 µL, and a 50-µL aliquot of the CMC solution including the wood particles was dispensed into each well of a 48-well microplate containing 500 µL/well of malt agar medium (45 g of 2.5% MA [Nissui, Tokyo, Japan], 1 g of yeast extract, 10 mg of chloramphenicol, and 1 L of distilled water). Eight microplates were prepared for each particle composite. The microplates were placed in polyethylene bags and incubated at room temperature (24−26 °C) under a 12/12-h light/dark cycle for 2 months. The plates were examined weekly under a stereomicroscope, and the colonies that presented Dacrymycetes characteristics (coloured white, purple, or yellow to orange and thin lanate or velvety in texture) were isolated and preserved in sealed vials containing 0.1% cornmeal agar (0.2% CMA; Nissui) + 1.25% malt agar (2.5% MA) medium (8.5 g of 0.2% CMA, 22.5 g of 2.5% MA, 1 g of yeast extract, and 1 L of distilled water). The isolated cultures are available from the Culture Collection of National Institute of Agrobiological Science (MAFF; Supplementary Table 1).

### DNA extraction/PCR/sequencing: fruiting bodies and cultures

Genomic DNA was extracted from fruiting bodies and cultured mycelia by using a Genomic DNA Extraction Kit Mini (Plant, RBC Bioscience, New Taipei City, Taiwan). The primers LR0R/LR5 (https://sites.duke.edu/vilgalyslab/rdna_primers_for_fungi/)^41^ were used to amplify a partial sequence of LSU rDNA. Each PCR mixture (10 µL) contained 1 µL of genomic DNA, 5 µL of EmeraldAmp PCR Master Mix (Takara Bio Inc., Shiga, Japan), 0.25 µL of each primer (10 µM), and 3.5 µL of Milli-Q water. The following thermocycling protocol was used: one cycle of 3 min at 94 °C, followed by 35 cycles of 30 s at 94 °C, 30 s at 51 °C, and 1 min at 72 °C, and a final 5-min cycle at 72 °C. The resulting PCR products were directly sequenced by SolGent (Daejeon, Korea). The obtained sequences were identified based on BLAST searches.

### DNA extraction/PCR/eDNA metabarcoding: decaying branches

A 50-mg sample of the dried wood powder obtained during implementation of the dilution-to-extinction method was placed in a 2-mL screw-tube containing zirconia beads (∅ 3 and 1 mm), and the beads were crushed by shaking at 2,500 rpm for 5 min in a bead crusher (µT-12, TAITEC, Saitama, Japan). DNA was extracted from the crushed wood powder by using the Genomic DNA Extraction Kit Mini (RBC Bioscience), and the extracted DNA was further purified using a MagExtractor (Toyobo, Osaka, Japan). The purified DNA was used as a template for the 1^st^ PCR, which was performed in four technical replicates per sample by using the following primers: ITS2D-Deg-TN 5′-*ACACTCTTTCCCTACACGACGCTCTTCCGATCT*NNNNNNTAGGRNTACCCGCTGAACTTAAGC-3′, designed for Dacrymycetes; and LR22-TN 5′-*GTGACTGGAGTTCAGACGTGTGCTCTTCCGATCT*NNNNNNCCTCACGGTACTTGTTCGCT-3′, based on the fungal universal primer LR22 (https://sites.duke.edu/vilgalyslab/rdna_primers_for_fungi/). The italicised and regular letters denote sequencing primers of MiSeq (Illumina, San Diego, CA, USA) and fungus-targeting primers, respectively. The six random bases (“N”) were used to enhance cluster separation on the flow cell during the initial base-call calibrations on MiSeq. Each PCR mixture (10 µL) contained 1 µL of DNA template, 5 µL of 2 × KAPA HiFi HS ReadyMix (KAPA Biosystems, Woburn, MA, USA), 0.25 µL of each primer (10 µM), and 3.5 µL of Milli-Q water. To suppress the production of chimeric sequences during PCR, we adopted a condition that combined slow temperature change (1 °C/s)^42^, high annealing temperature, and a small number of PCR cycles^43^. The thermocycling protocol was as follows: one cycle of 3 min at 95 °C, followed by six cycles of 20 s at 98 °C, 15 s from 67 to 62 °C, and 15 s at 72 °C, followed by 24 cycles of 20 s at 98 °C, 15 s at 62 °C, and 15 s at 72 °C, and a final 5-min cycle at 72 °C. The 1^st^ PCR product was purified using Agencourt AMPure XP (Beckman Coulter, Brea, CA, USA).

The 2^nd^ PCR amplified the 1^st^ PCR amplicons by using the prime rs (forward) 5′-*AATGATACGGCACCACCGAGATCTACAC*XXXXXXXXTCGTCGGCAGCGTCAGATGTGTATAAGAGACAG-3′ a nd (reverse) 5′-*CAAGCAGAAGACGGCATACGAGAT*XXXXXXXXGTCTCGTGGGCTCGGAGATGTGTATAA AGACAG-3′. The italicised and regular letters denote the MiSeqP5/P7 adapter and sequencing primers, respectively. The eight bases denoted by “X” represent dual-index sequences inserted to identify distinct samples^44^. The 2^nd^ PCR was performed using a 12-µL reaction mixture containing 1 µL of DNA template, 6 µL of KAPA HiFi HS ReadyMix, 1.4 µL of each primer (2.5 µM), and 2.2 µL of Milli-Q water. The following PCR conditions were used: one cycle of 3 min at 95 °C, followed by 12 cycles of 20 s at 98 °C, 15 s at 72 °C, and a final extension for 5 min at 72 °C.

The indexed 2^nd^ PCR amplicons were pooled to prepare a library for MiSeq sequencing. The volume of each sample added to the library was adjusted to normalise the concentration of each 2^nd^ PCR product. The pooled library was purified using Agencourt AMPure XP, and then target-sized DNAs in the purified library (~500 bp) were excised using E-Gel SizeSelect (Thermo Fisher Scientific, Waltham, MA, USA). The double-stranded DNA concentration of the library was adjusted to 4 nM by using Milli-Q water, and the DNA sample was applied to the Illumina MiSeq platform at Ryukoku University, Japan, by using MiSeq Reagent Kit V2 (500 cycles). Sequence data were deposited in the Sequence Read Archive of the DDBJ under an accession number: DRA008716 (ftp://ftp.ddbj.nig.ac.jp/ddbj_database/dra/fastq/DRA008/DRA008716/).

The procedures used for bioinformatics and data analyses followed those described previously^45^. By using the bcl2fastq programme provided by Illumina, the raw MiSeq data were converted into FASTQ files, which were demultiplexed using the commands implemented in Claident pipeline (https://www.claident.org/)^46^. The forward and reverse sequences were then merged. The total 6,918,978 reads (n = 288) were assembled using Claident v0.2.2016.07.05. Subsequently, short reads (<150 bp) were removed, and sequencing errors were removed using algorithms in CD-HIT-OTU^47^. The remaining sequences were assembled at a threshold similarity of 97%^4^, and the resulting consensus sequences represented molecular OTUs. For OTUs of clustered eDNA, sequences obtained from fruiting bodies and isolated cultures were mapped with a similarity of 97%. In preliminary analyses, we confirmed that the results of mapping were the same as those of *de novo* clustering. Following this process, an OTU table (i.e. a matrix of OTUs and samples with sequence reads in each cell entry) was generated. To eliminate putative contaminations, cell entries of the OTU table with ≤ 9 reads were removed. For each sample, only the OTUs found in all four technical replicates were used for the following analysis to reduce the possibility of false-positive detection. To eliminate chimeric OTUs, the obtained sequences were tested based on the results of the chimera-finding algorithm UCHIME^48^ by using reference sequences in the SILVA database, BLAST search after dividing the sequences into the first half and second half^24^, and preliminary phylogenetic analyses. Subsequently, the suspected chimeric sequences were removed from further analysis.

### Molecular phylogenetic analysis

The dataset of LSU rDNA sequences was assembled to include the representative sequences of each OTU obtained using each detection method and the sequences of Dacrymycetes downloaded from the National Center for Biotechnology Information (NCBI, 30 June 2019) regarding previous studies^4,29,32,49,50^. Multiple alignments were generated using MAFFT 7 (https://mafft.cbrc.jp/alignment/server/)^51^ with the G-INS-i option. Ambiguously aligned sequence regions such as significant gaps were manually removed before subsequent analysis. Phylogenetic trees were estimated using RAxML v. 8.2.10^52^ under a GTRGAMMA model. Maximum-likelihood bootstrap percentages (MLBPs) and a tree were obtained by concurrently running rapid bootstrap analyses of 1,000 pseudoreplicates followed by a search for the most likely tree. The sequences determined in this study and used for the phylogenetic analysis were deposited in the DDBJ (Supplementary Table 1). The aligned dataset was uploaded to TreeBASE under ID: S24901 (http://purl.org/phylo/treebase/phylows/study/TB2:S24901).

### Statistical analyses

All statistical analyses were performed using R 3.5.3^53^. Statistical significance, α, was set as 0.05. Based on the presence/absence data of the OTUs detected using each method at each sampling event, sample-based rarefaction curves were generated using “iNEXT” package^54^ with the first Hill number (q = 0). We analysed the effects of the following explanatory variables that were likely to affect the richness and composition of OTUs: the detection method (fruiting-body collection, culture isolation, or eDNA analysis), forest age of plots (45 or 70 years), decomposition stage of branches (II, III, or IV), season of sampling events (May, July, September, or November), and year (2016, 2017, or 2018). A GLM with a Poisson distribution was applied to determine the factors affecting the OTU richness. To test the differences in detection methods, multiple comparisons of the OTU richness between methods were performed using the post-hoc Tukey’s test by using the “multicomp” package^55^. Additional GLMs were implemented to evaluate the effects of the other explanatory variables on OTU richness and phylogenetic diversity in each method. A model selection procedure was implemented based on AICc. To evaluate the phylogenetic diversity, SESs were calculated for MPD and MNTD^56^ by using the “picante” package^57^. This was only performed for the eDNA metabarcoding data as the other methods detected only 0–1 OTU in most samples and the phylogenetic diversity could not be calculated. Subsequently, model selection was performed using a Gaussian distribution. To evaluate the effects of explanatory variables on OTU composition, a PERMANOVA was performed using two dissimilarity indices calculated for the complete dataset: the Raup-Crick metric with the “vegan” package^58^ and the Unifrac metric with the “picante” package. The Raup-Crick metric is weakly affected by the species-richness gradient among sampling units, and the Unifrac metric quantifies the relative relatedness of OTUs by incorporating phylogenetic distances. As the number of OTUs detected using the culture method was small, dissimilarity was not reliably calculated.

## Supplementary information

Supplementary Information.

Supplementary Data 1.

Supplementary Data 2.

## Data Availability

Tables of OTUs and environmental variables used for all graphs and statistical analyses are available online as Supplementary Data Files. The DNA sequence and alignment data are publicly available at DDBJ (LC492144–LC492294, DRA008716) and TreeBASE (ID: S24901), respectively.
