# Supplementary material for: Complementary molecular methods reveal comprehensive phylogenetic diversity integrating inconspicuous lineages of early-diverged wood-decaying mushrooms
Source: Sci Rep. 2020 Feb 20;10:3057. doi: 10.1038/s41598-020-59620-0 (PMC7033186; doi:10.1038/s41598-020-59620-0)
Supplement: Supplementary file 1 — Supplementary Information. [file 41598_2020_59620_MOESM1_ESM.pdf]

SUPPLEMENTARY MATERIAL

Complementary molecular methods reveal comprehensive phylogenetic diversity integrating inconspicuous lineages of early-diverged wood-decaying mushrooms

Takashi Shirouzu[1]*, Shunsuke Matsuoka[2], Hideyuki Doi[2], Nobuaki Nagata[3], Masayuki Ushio[4,5,6] & Kentaro Hosaka[7]

[1] Graduate School of Bioresources, Mie University, 1577 Kurima-machiya, Tsu, Mie 514-8507, Japan.

[2] Graduate School of Simulation Studies, University of Hyogo, 7-1-28 Minatojima-minamimachi, Chuo-ku, Kobe, Hyogo 650-0047, Japan.

[3] Collection Center, National Museum of Nature and Science, 4-1-1 Amakubo, Tsukuba, Ibaraki 305-0005, Japan.

[4] Hakubi Center, Kyoto University, Kyoto 606-8501, Japan

[5] Center for Ecological Research, Kyoto University, Hirano 2-509-3, Otsu, Shiga 520-2113, Japan.

[6] PRESTO, Japan Science and Technology Agency, Kawaguchi 332-0012, Japan

[7] Department of Botany, National Museum of Nature and Science, 4-1-1 Amakubo, Tsukuba, Ibaraki 305-0005, Japan.

* Corresponding author: shirouzy@gmail.com

Supplementary Figure Legend

Supplementary Fig. 1 Phylogenetic tree of Agaricomycotina estimated by using RAxML with large subunit ribosomal DNA (LSU rDNA) sequences. Thick branches indicate maximum-likelihood bootstrap percentages (MLBP) ≥ 80%. Analysis was conducted in the same manner as for the phylogenetic tree of Dacrymycetes (Fig. 2).

Supplementary Tables

Supplementary Table 1 Specimens, cultures, and accession numbers of sequences

Supplementary Table 2 All results of AICc-based model selection for the effects of environmental factors on OTU richness detected according to fruiting-body collection

Supplementary Table 3 All results of AICc-based model selection for the effects of environmental factors on OTU richness detected according to culture isolation

Supplementary Table 4 All results of AICc-based model selection for the effects of environmental factors on OTU richness detected according to eDNA analysis

Supplementary Table 5 All results of AICc-based model selection for the effects of environmental factors on MPD (SES) detected according to eDNA analysis

Supplementary Table 6 All results of AICc-based model selection for the effects of environmental factors on MNTD (SES) detected according to eDNA analysis

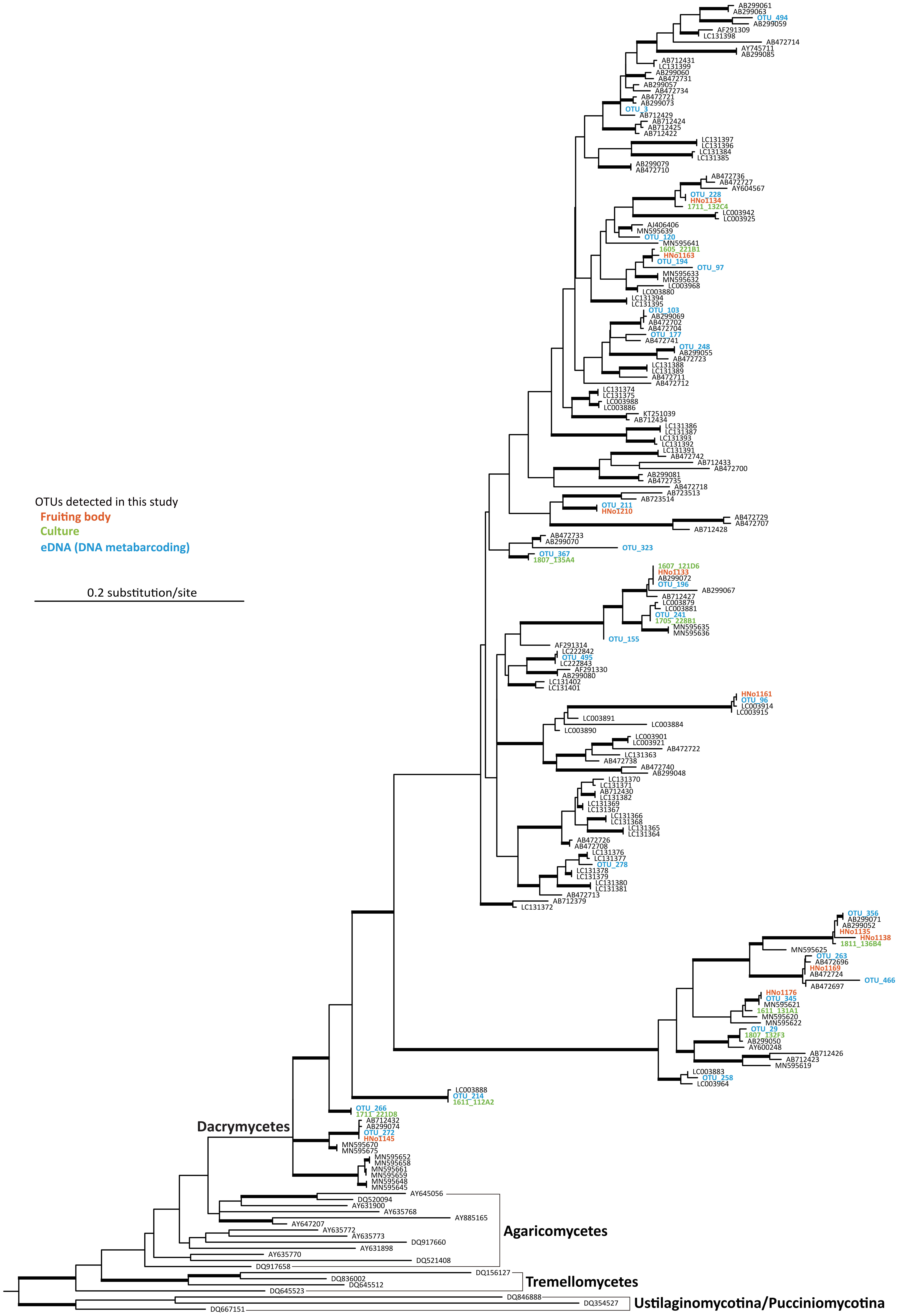

Supplementary Fig. 1 Phylogenetic tree of Agaricomycotina estimated by using RAxML with large subunit ribosomal DNA (LSU rDNA) sequences. Thick branches indicate maximum-likelihood bootstrap percentages (MLBP) ≥ 80%. Analysis was conducted in the same manner as for the phylogenetic tree of Dacrymycetes (Fig. 2).

Supplementary Table 1 Specimens, cultures, and accession numbers of sequences

Sample name	Specimen or sample no.[1]	Culture no.[2]	Related eDNA (similarity threshold of 97%)	Collected month	Plot	Decomposition stage	DDBJ accession no. LSU[3]
**Present study**							
**Fruiting body**							
HNo1133	TNS-F-88721	-	OTU_196	Jul. 2016	Plot 1	III	LC492144
HNo1134	TNS-F-88722	-	OTU_228	Jul. 2016	Plot 1	III	LC492145
HNo1135	TNS-F-88723	-	OTU_356	Jul. 2016	Plot 1	III	LC492146
HNo1136	TNS-F-88724	-	OTU_196	Jul. 2016	Plot 1	III	LC492147
HNo1137	TNS-F-88725	-	OTU_196	Jul. 2016	Plot 1	III	LC492148
HNo1138	TNS-F-88726	-	-	Jul. 2016	Plot 1	II	LC492149
HNo1139	TNS-F-88727	-	OTU_196	Jul. 2016	Plot 1	III	LC492150
HNo1140	TNS-F-88728	-	OTU_356	Jul. 2016	Plot 1	III	LC492151
HNo1141	TNS-F-88729	-	OTU_196	Jul. 2016	Plot 1	III	LC492152
HNo1144	TNS-F-88730	-	OTU_196	Jul. 2016	Plot 2	III	LC492153
HNo1145	TNS-F-88731	-	OTU_272	Jul. 2016	Plot 2	III	LC492154
HNo1146	TNS-F-88732	-	OTU_272	Jul. 2016	Plot 2	III	LC492155
HNo1147	TNS-F-88733	-	OTU_196	Jul. 2016	Plot 2	III	LC492156
HNo1148	TNS-F-88734	-	OTU_356	Jul. 2016	Plot 2	III	LC492157
HNo1149	TNS-F-88735	-	OTU_196	Jul. 2016	Plot 2	III	LC492158
HNo1150	TNS-F-88736	-	OTU_196	Jul. 2016	Plot 2	III	LC492159
HNo1151	TNS-F-88737	-	OTU_196	Jul. 2016	Plot 2	II	LC492160
HNo1152	TNS-F-88738	-	OTU_196	Jul. 2016	Plot 2	II	LC492161
HNo1153	TNS-F-88739	-	OTU_196	Jul. 2016	Plot 2	II	LC492162
HNo1154	TNS-F-88740	-	OTU_196	Sep. 2016	Plot 1	III	LC492163
HNo1155	TNS-F-88741	-	OTU_196	Sep. 2016	Plot 1	II	LC492164
HNo1156	TNS-F-88742	-	OTU_356	Sep. 2016	Plot 1	III	LC492165
HNo1157	TNS-F-88743	-	OTU_272	Sep. 2016	Plot 1	IV	LC492166
HNo1158	TNS-F-88744	-	OTU_272	Sep. 2016	Plot 1	IV	LC492167
HNo1159	TNS-F-88745	-	OTU_356	Sep. 2016	Plot 2	III	LC492168
HNo1161	TNS-F-88746	-	OTU_96	Sep. 2016	Plot 2	II	LC492169
HNo1162	TNS-F-88747	-	OTU_96	Sep. 2016	Plot 2	II	LC492170
HNo1163	TNS-F-88748	-	OTU_194	Sep. 2016	Plot 2	III	LC492171
HNo1164	TNS-F-88749	-	OTU_196	Sep. 2016	Plot 2	III	LC492172
HNo1165	TNS-F-88750	-	OTU_196	Nov. 2016	Plot 1	III	LC492173
HNo1166	TNS-F-88751	-	OTU_196	Nov. 2016	Plot 1	III	LC492174
HNo1167	TNS-F-88752	-	OTU_196	Nov. 2016	Plot 1	IV	LC492175
HNo1168	TNS-F-88753	-	OTU_356	Nov. 2016	Plot 2	IV	LC492176
HNo1169	TNS-F-88754	-	OTU_263	Nov. 2016	Plot 2	IV	LC492177
HNo1170	TNS-F-88755	-	OTU_196	Nov. 2016	Plot 2	III	LC492178
HNo1175	TNS-F-88756	-	OTU_196	May 2017	Plot 2	III	LC492179
HNo1176	TNS-F-88757	-	OTU_345	Jul. 2017	Plot 1	III	LC492180
HNo1177	TNS-F-88758	-	OTU_196	Jul. 2017	Plot 1	III	LC492181
HNo1178	TNS-F-88759	-	OTU_196	Jul. 2017	Plot 1	III	LC492182
HNo1179	TNS-F-88760	-	OTU_194	Jul. 2017	Plot 1	III	LC492183
HNo1180	TNS-F-88761	-	OTU_272	Jul. 2017	Plot 1	IV	LC492184
HNo1181	TNS-F-88762	-	OTU_356	Jul. 2017	Plot 1	IV	LC492185
HNo1182	TNS-F-88763	-	OTU_356	Jul. 2017	Plot 1	III	LC492186
HNo1183	TNS-F-88764	-	OTU_272	Jul. 2017	Plot 1	III	LC492187
HNo1185	TNS-F-88765	-	OTU_194	Jul. 2017	Plot 1	III	LC492188
HNo1186	TNS-F-88766	-	OTU_194	Jul. 2017	Plot 2	III	LC492189
HNo1187	TNS-F-88767	-	OTU_356	Jul. 2017	Plot 2	III	LC492190
HNo1188	TNS-F-88768	-	OTU_356	Jul. 2017	Plot 2	III	LC492191
HNo1189	TNS-F-88769	-	OTU_196	Jul. 2017	Plot 2	III	LC492192
HNo1190	TNS-F-88770	-	OTU_196	Jul. 2017	Plot 2	III	LC492193
HNo1192	TNS-F-88771	-	OTU_196	Sep. 2017	Plot 2	III	LC492194
HNo1195	TNS-F-88772	-	OTU_196	Nov. 2017	Plot 1	III	LC492195
HNo1202	TNS-F-88773	-	OTU_196	Nov. 2017	Plot 2	II	LC492196
HNo1206	TNS-F-88774	-	OTU_196	Jul. 2018	Plot 2	III	LC492197
HNo1207	TNS-F-88775	-	OTU_196	Jul. 2018	Plot 2	III	LC492198
HNo1210	TNS-F-88776	-	OTU_211	Sep. 2018	Plot 1	III	LC492199
HNo1212	TNS-F-88777	-	OTU_263	Sep. 2018	Plot 1	III	LC492200
HNo1213	TNS-F-88778	-	OTU_196	Sep. 2018	Plot 1	III	LC492201
HNo1214	TNS-F-88779	-	OTU_196	Sep. 2018	Plot 1	III	LC492202
HNo1215	TNS-F-88780	-	OTU_345	Sep. 2018	Plot 1	II	LC492203
HNo1216	TNS-F-88781	-	OTU_356	Sep. 2018	Plot 1	III	LC492204
HNo1217	TNS-F-88782	-	OTU_272	Sep. 2018	Plot 1	III	LC492205
HNo1218	TNS-F-88783	-	OTU_194	Sep. 2018	Plot 1	II	LC492206
HNo1219	TNS-F-88784	-	OTU_194	Sep. 2018	Plot 2	III	LC492207
HNo1220	TNS-F-88785	-	OTU_196	Sep. 2018	Plot 2	III	LC492208
HNo1221	TNS-F-88786	-	OTU_194	Sep. 2018	Plot 2	II	LC492209
HNo1222	TNS-F-88787	-	OTU_194	Sep. 2018	Plot 2	II	LC492210
HNo1223	TNS-F-88788	-	OTU_196	Sep. 2018	Plot 2	III	LC492211
HNo1224	TNS-F-88789	-	OTU_196	Sep. 2018	Plot 2	III	LC492212
HNo1225	TNS-F-88790	-	OTU_194	Sep. 2018	Plot 2	II	LC492213
HNo1226	TNS-F-88791	-	OTU_196	Nov. 2018	Plot 1	III	LC492214
HNo1227	TNS-F-88792	-	OTU_196	Nov. 2018	Plot 1	III	LC492215
HNo1228	TNS-F-88793	-	OTU_196	Nov. 2018	Plot 1	III	LC492216
HNo1229	TNS-F-88794	-	OTU_196	Nov. 2018	Plot 2	III	LC492217
HNo1230	TNS-F-88795	-	OTU_196	Nov. 2018	Plot 2	IV	LC492218
**Culture**							
1605_115B6	-	-[4]	OTU_194	May 2016	Plot 1	II	LC492219
1605_221B1	-	-[4]	OTU_194	May 2016	Plot 2	III	LC492220
1605_224C1	-	-[4]	OTU_194	May 2016	Plot 2	III	LC492221
1605_225F6	-	MAFF 247103	OTU_194	May 2016	Plot 2	III	LC492222
1605_227D5	-	-[4]	OTU_194	May 2016	Plot 2	III	LC492223
1605_227D7	-	-[4]	OTU_194	May 2016	Plot 2	III	LC492224
1607_121D6	-	-[4]	OTU_196	Jul. 2016	Plot 1	III	LC492225
1611_112A2	-	MAFF 247104	OTU_214	Nov. 2016	Plot 1	II	LC492226
1611_131A1	-	-[4]	OTU_345	Nov. 2016	Plot 1	IV	LC492227
1611_225B6	-	-[4]	OTU_194	Nov. 2016	Plot 2	III	LC492228
1611_226A2	-	-[4]	OTU_194	Nov. 2016	Plot 2	III	LC492229
1611_226B4	-	-[4]	OTU_194	Nov. 2016	Plot 2	III	LC492230
1611_226E1	-	-[4]	OTU_194	Nov. 2016	Plot 2	III	LC492231
1611_235C3	-	MAFF 247105	OTU_196	Nov. 2016	Plot 2	IV	LC492232

1705_133F2	-	MAFF 247106	OTU_194	May 2017	Plot 1	IV	LC492233
1705_211A1	-	MAFF 247107	OTU_196	May 2017	Plot 2	II	LC492234
1705_211D8	-	MAFF 247108	OTU_196	May 2017	Plot 2	II	LC492235
1705_217A6	-	MAFF 247150	OTU_196	May 2017	Plot 2	II	LC492236
1705_218C5	-	-[4]	OTU_194	May 2017	Plot 2	II	LC492237
1705_228B1	-	MAFF 247109	OTU_241	May 2017	Plot 2	III	LC492238
1707_114F1	-	MAFF 247110	OTU_194	Jul. 2017	Plot 1	II	LC492239
1707_117C6	-	-[4]	OTU_194	Jul. 2017	Plot 1	II	LC492240
1709_217F7	-	MAFF 247111	OTU_194	Sep. 2017	Plot 2	II	LC492241
1711_132C4	-	-[4]	OTU_228	Nov. 2017	Plot 1	IV	LC492242
1711_135D1	-	-[4]	OTU_241	Nov. 2017	Plot 1	IV	LC492243
1711_136D7	-	-[4]	OTU_228	Nov. 2017	Plot 1	IV	LC492244
1711_218A1	-	MAFF 247112	OTU_214	Nov. 2017	Plot 2	II	LC492245
1711_221D8	-	MAFF 247161	OTU_266	Nov. 2017	Plot 2	III	LC492246
1805_127F7	-	MAFF 247113	OTU_194	May 2018	Plot 1	III	LC492247
1805_138B8	-	MAFF 247151	OTU_266	May 2018	Plot 1	IV	LC492248
1807_132F3	-	MAFF 247114	OTU_29	Jul. 2018	Plot 1	IV	LC492249
1807_135A4	-	MAFF 247115	OTU_367	Jul. 2018	Plot 1	IV	LC492250
1807_137D8	-	MAFF 247116	OTU_194	Jul. 2018	Plot 1	IV	LC492251
1809_121D5	-	MAFF 247117	OTU_194	Sep. 2018	Plot 1	III	LC492252
1809_121E1	-	MAFF 247118	OTU_194	Sep. 2018	Plot 1	III	LC492253
1809_123C2	-	MAFF 247119	OTU_194	Sep. 2018	Plot 1	III	LC492254
1809_123D8	-	MAFF 247120	OTU_194	Sep. 2018	Plot 1	III	LC492255
1809_124B8	-	MAFF 247121	OTU_194	Sep. 2018	Plot 1	III	LC492256
1809_125E1	-	MAFF 247122	OTU_194	Sep. 2018	Plot 1	III	LC492257
1809_126B7	-	MAFF 247123	OTU_194	Sep. 2018	Plot 1	III	LC492258
1809_127E1	-	MAFF 247124	OTU_194	Sep. 2018	Plot 1	III	LC492259
1809_127E3	-	MAFF 247125	OTU_194	Sep. 2018	Plot 1	III	LC492260
1809_128C2	-	MAFF 247126	OTU_194	Sep. 2018	Plot 1	III	LC492261
1809_133F6	-	MAFF 247127	OTU_194	Sep. 2018	Plot 1	IV	LC492262
1809_137F1	-	MAFF 247128	OTU_194	Sep. 2018	Plot 1	IV	LC492263
1809_233D6	-	MAFF 247129	OTU_241	Sep. 2018	Plot 2	IV	LC492264
1811_124B4	-	MAFF 247130	OTU_194	Nov. 2018	Plot 1	III	LC492265
1811_131E7	-	MAFF 247131	OTU_194	Nov. 2018	Plot 1	IV	LC492266
1811_136B4	-	MAFF 247132	OTU_356	Nov. 2018	Plot 1	IV	LC492267
**eDNA**							
OTU_3	-	-	OTU_3	May 2016, Sep. 2018	Plot 1, 2	II, III, IV	LC492268
OTU_29	-	-	OTU_29	Nov. 2017, Jul. 2018	Plot 1	II, IV	LC492269
OTU_96	-	-	OTU_96	May 2016, Nov. 2018	Plot 1, 2	II	LC492270
OTU_97	-	-	OTU_97	Nov. 2016, Sep., Nov. 2018	Plot 1, 2	III, IV	LC492271
OTU_103	-	-	OTU_103	Nov. 2018	Plot 1	II	LC492272
OTU_120	-	-	OTU_120	Nov. 2016, May, Sep., Nov. 2017, May, Sep. 2018	Plot 1, 2	II, III	LC492273
OTU_155	-	-	OTU_155	Jul. 2016, May, Sep. 2017, Sep., Nov. 2018	Plot 1, 2	II, III	LC492274
OTU_177	-	-	OTU_177	May, Jul., Sep. 2016, May, Sep., Nov. 2017, May, Jul., Sep., Nov. 2018	Plot 1, 2	II, III	LC492275
OTU_194	-	-	OTU_194	May, Jul.,Sep., Nov. 2016, May, Jul., Sep., Nov. 2017, May, Jul., Sep., Nov. 2018	Plot 1, 2	II, III, IV	LC492276
OTU_196	-	-	OTU_196	May, Jul.,Sep., Nov. 2016, May, Jul., Sep., Nov. 2017, May, Jul., Sep., Nov. 2018	Plot 1, 2	II, III, IV	LC492277
OTU_211	-	-	OTU_211	May, Jul., Sep. 2016, May, Jul., Sep., Nov. 2017, May, Jul., Nov. 2018	Plot 1, 2	II, III, IV	LC492278
OTU_214	-	-	OTU_214	May, Jul.,Sep., Nov. 2016, May, Jul., Sep., Nov. 2017, May, Jul., Sep., Nov. 2018	Plot 1, 2	II, III, IV	LC492279
OTU_228	-	-	OTU_228	May, Jul.,Sep., Nov. 2016, May, Jul., Sep., Nov. 2017, May, Jul. 2018	Plot 1, 2	II, III, IV	LC492280
OTU_241	-	-	OTU_241	May, Jul., Sep. Nov. 2016, Jul., Sep., Nov. 2017, May, Jul., Sep., Nov. 2018	Plot 1, 2	II, III, IV	LC492281
OTU_248	-	-	OTU_248	May, Sep., Nov. 2017, Sep. 2018	Plot 1, 2	II, III, IV	LC492282
OTU_258	-	-	OTU_258	May, Jul., Nov. 2016, May, Jul., Sep., Nov. 2017, May, Jul., Nov. 2018	Plot 1, 2	II, III, IV	LC492283
OTU_263	-	-	OTU_263	May, Jul., Sep., Nov. 2016, May, Jul., Nov. 2017, May, Jul., Sep., Nov. 2018	Plot 1, 2	II, III, IV	LC492284
OTU_266	-	-	OTU_266	May, Jul.,Sep., Nov. 2016, May, Jul., Sep., Nov. 2017, May, Jul., Sep., Nov. 2018	Plot 1, 2	II, III, IV	LC492285
OTU_272	-	-	OTU_272	May, Jul.,Sep., Nov. 2016, May, Jul., Sep., Nov. 2017, May, Jul., Sep., Nov. 2018	Plot 1, 2	II, III, IV	LC492286
OTU_278	-	-	OTU_278	May, Jul., Sep. 2016, May, Sep., Nov. 2017, Jul., Nov. 2018	Plot 1, 2	II, III, IV	LC492287
OTU_323	-	-	OTU_323	May 2016, Nov. 2017	Plot 1, 2	II, III	LC492288
OTU_345	-	-	OTU_345	May, Jul., Sep., Nov. 2016, Nov. 2017, May, Sep. 2018	Plot 1, 2	III, IV	LC492289
OTU_356	-	-	OTU_356	May, Jul., Sep., Nov. 2016, May, Jul., Nov. 2017, May, Jul., Sep., Nov. 2018	Plot 1, 2	II, III, IV	LC492290
OTU_367	-	-	OTU_367	Nov. 2017	Plot 2	IV	LC492291
OTU_466	-	-	OTU_466	May, Sep. 2016, Jul., Nov. 2017, May, Jul., Sep., Nov. 2018	Plot 1, 2	II, III, IV	LC492292
OTU_494	-	-	OTU_494	May 2016	Plot 2	II	LC492293
OTU_495	-	-	OTU_495	May 2016	Plot 1	III	LC492294
**Previous studies**							
Calocera arborea	INPA 241457	-	-	-	-	-	AB723513
C. arborea	INPA 241458	-	-	-	-	-	AB723514
C. cornea	TNS-F-21061	MAFF 241186	-	-	-	-	AB472722
C. cornea	-	CBS 124.84	-	-	-	-	AB472738
C. cornea	PDD 107847	ICMP 21223	-	-	-	-	LC131363
C. fusca	PDD 107930	-	-	-	-	-	LC131364
C. fusca	PDD 107972	ICMP 21238	-	-	-	-	LC131365
C . cf. *guepinioides*	PDD 107929	ICMP 21231	-	-	-	-	LC131369
C . cf. *guepinioides*	PDD 105033	ICMP 20502	-	-	-	-	LC131367
C . cf. *guepinioides*	PDD 107981	ICMP 21240	-	-	-	-	LC131371
C . cf. *guepinioides*	PDD 107969	ICMP 21236	-	-	-	-	LC131370
C . cf. *guepinioides*	PDD 107874	ICMP 21226	-	-	-	-	LC131368
C . cf. *guepinioides*	PDD 105005	ICMP 20480	-	-	-	-	LC131366
C. lutea	-	CBS 291.82	-	-	-	-	AB712379
C. lutea	PDD 107841	ICMP 21221	-	-	-	-	LC131372
C. pedicellata	PDD 107830	-	-	-	-	-	LC131374
C. pedicellata	PDD 107925	ICMP 21230	-	-	-	-	LC131375
C. viscosa	TNS-F-15704	MAFF 240119	-	-	-	-	AB299048
C. viscosa	-	CBS 292.82	-	-	-	-	AB472740
Cerinomyces albosporus	TNS-F-15706	MAFF 240121	-	-	-	-	AB299050
C. canadensis	TNS-F-21034	MAFF 241162	-	-	-	-	AB472696
C. canadensis	TNS-F-21035	MAFF 241163	-	-	-	-	AB472697
C. ceraceus	-	HHB-8969	-	-	-	-	AB712422
C. crustulinus	-	TUFC 30545	-	-	-	-	AB712423
C. crustulinus	-	-	-	-	-	-	AY600248
C. grandinioides	-	HHB-6908	-	-	-	-	AB712424
C. lagerheimii	-	RLG-13487	-	-	-	-	AB712425
C. pallidus	TNS-F-21064	-	-	-	-	-	AB472724
C. pallidus	-	FP150848	-	-	-	-	AB712426
Cerinomyces sp.	O 247959	-	-	-	-	-	MN595619
Dacrymyces adpressus	TNS-F-21069	MAFF 241191	-	-	-	-	AB472729
D. adpressus	TNS-F-21045	MAFF 241172	-	-	-	-	AB472707

D. ancyleus	TNS-F-21051	MAFF 241177	-	-	-	-	AB472713
D. capitatus	-	CBS 293.82	-	-	-	-	AB472741
D. capitatus	TNS-F-15709	MAFF 240124	-	-	-	-	AB299055
D. capitatus	TNS-F-21062	MAFF 241187	-	-	-	-	AB472723
D. chrysocomus	-	CBS 280.84	-	-	-	-	AB712427
D. chrysospermus	TNS-F-21060	MAFF 241185	-	-	-	-	AB472721
D. chrysospermus	TNS-F-15712	MAFF 240127	-	-	-	-	AB299073
D. citrinus	PDD 107915	ICMP 21227	-	-	-	-	LC131376
D. citrinus	PDD 107979	ICMP 21239	-	-	-	-	LC131377
D. cylindricus	PDD 105052	ICMP 20517	-	-	-	-	LC131378
D. cylindricus	PDD 107989	-	-	-	-	-	LC131379
D. cyrtosporus	PDD 107980	-	-	-	-	-	LC131381
D. cyrtosporus	PDD 107952	-	-	-	-	-	LC131380
D. dendrocalami	TNS-F-38903	TUFC 13914	-	-	-	-	AB712428
D. dictyosporus	-	HHB-8618	-	-	-	-	AB712429
D. estonicus	UPS F-940137	-	-	-	-	-	MN595632
D. estonicus	UPS F-940138	-	-	-	-	-	MN595633
D. flabelliformis	PDD 76696	HHB-18308	-	-	-	-	AB712430
D. flabelliformis	PDD 107863	ICMP 21225	-	-	-	-	LC131382
D. intermedius	PDD 107939	ICMP 21232	-	-	-	-	LC131385
D. intermedius	PDD 107851	ICMP 21224	-	-	-	-	LC131384
D. lacrymalis	TNS-F-15719	MAFF 240134	-	-	-	-	AB299069
D. lacrymalis	TNS-F-21040	MAFF 241167	-	-	-	-	AB472702
D. lacrymalis	TNS-F-21042	MAFF 241169	-	-	-	-	AB472704
D. longistipitatus	PDD 107997	ICMP 21242	-	-	-	-	LC131387
D. longistipitatus	PDD 107996	ICMP 21241	-	-	-	-	LC131386
D. microsporus	TNS-F-21049	MAFF 241175	-	-	-	-	AB472711
D. microsporus	TNS-F-21050	MAFF 241176	-	-	-	-	AB472712
D. cf. *microsporus*	PDD 104992	ICMP 20466	-	-	-	-	LC131388
D. cf. *microsporus*	PDD 104993	ICMP 20467	-	-	-	-	LC131389
D. minor	TNS-F-15721	MAFF 240136	-	-	-	-	AB299063
D. minor	TNS-F-15720	MAFF 240135	-	-	-	-	AB299059
D. minutus	TNS-F-21073	-	-	-	-	-	AB472733
D. minutus	TNS-F-15722	MAFF 240137	-	-	-	-	AB299070
D. novae-zelandiae	TNS-F-21038	MAFF 241165	-	-	-	-	AB472700
D. novae-zelandiae	PDD 107953	ICMP 21235	-	-	-	-	LC131391
D. novae-zelandiae	-	CBS 295.82	-	-	-	-	AB472742
D. ovisporus	UPS F-940139	-	-	-	-	-	MN595635
D. ovisporus	UPS F-940140	-	-	-	-	-	MN595636
D. pachysporus	PDD 107916	ICMP 21228	-	-	-	-	LC131393
D. pachysporus	PDD 105004	ICMP 20479	-	-	-	-	LC131392
D. parastenosporus	PDD 104960	ICMP 20433	-	-	-	-	LC131394
D. parastenosporus	PDD 104963	ICMP 20436	-	-	-	-	LC131395
D. pezizoides	TUMH 50290	TUFC 14269	-	-	-	-	LC386895
D. pezizoides	TNS-F-54907	MAFF 246756	-	-	-	-	LC386892
D. pinacearum	TNS-F-21056	MAFF 241182	-	-	-	-	AB472718
D. punctiformis	TNS-F-15723	MAFF 240138	-	-	-	-	AB299052
D. punctiformis	TNS-F-15725	MAFF 240140	-	-	-	-	AB299071
D. puniceus	TNS-F-15711	MAFF 240126	-	-	-	-	AB299057
D. puniceus	TNS-F-21074	MAFF 241195	-	-	-	-	AB472734
D. san-augustinii	TNS-F-15726	MAFF 240141	-	-	-	-	AB299081
D. san-augustinii	TNS-F-21075	MAFF 241196	-	-	-	-	AB472735
D. stenosporus	PDD 107970	ICMP 21237	-	-	-	-	LC131397
D. stenosporus	PDD 105018	ICMP 20488	-	-	-	-	LC131396
D. stillatus	FO28136	-	-	-	-	-	AF291309
D. stillatus	TNS-F-15727	MAFF 240142	-	-	-	-	AB299061
D. stillatus	TNS-F-21052	MAFF 241178	-	-	-	-	AB472714
D. cf. *stillatus*	PDD 105038	ICMP 20505	-	-	-	-	LC131398
D. subalpinus	TNS-F-21071	MAFF 241193	-	-	-	-	AB472731
D. subalpinus	TNS-F-15730	MAFF 240145	-	-	-	-	AB299060
D. subantarcticensis	PDD 107948	ICMP 21234	-	-	-	-	LC131399
D. subantarcticensis	PDD 76679	HHB-18220	-	-	-	-	AB712431
D. subarcticus	TNS-F-21076	-	-	-	-	-	AB472736
D. subarcticus	TNS-F-21067	-	-	-	-	-	AB472727
D. tortus s.l. 1	UPS F-940777	-	-	-	-	-	MN595620
D. tortus s.l. 2	UPS F-940948	-	-	-	-	-	MN595621
D. tortus s.l. 3	UPS F-941016	-	-	-	-	-	MN595622
D. tortus s.l. 4	UPS F-941020	-	-	-	-	-	MN595625
D. variisporus	TNS-F-15733	MAFF 240148	-	-	-	-	AB299072
D. variisporus	TNS-F-15732	MAFF 240147	-	-	-	-	AB299067
Dacryomitra pusilla	FO38346	-	-	-	-	-	AJ406406
D. pusilla	UPS F-176774	-	-	-	-	-	MN595639
Dacryonaema macnabbii	UPS F-940991	-	-	-	-	-	MN595658
D. macnabbii	UPS F-940952	-	-	-	-	-	MN595652
D. macrosporum	O 160045	-	-	-	-	-	MN595659
D. macrosporum	UPS F-941001	-	-	-	-	-	MN595661
D. rufum	UPS F-941003	-	-	-	-	-	MN595645
D. rufum	O 295528	-	-	-	-	-	MN595648
Dacryopinax elegans	-	HHB-18731	-	-	-	-	AB712433
D. indacochea	-	CRM-72	-	-	-	-	AB712434
D. primogenitus	MIN862738	CBS 140892	-	-	-	-	KT251039
D. spathularia	TNS-F-15736	MAFF 240151	-	-	-	-	AB299079
D. spathularia	TNS-F-21048	MAFF 241174	-	-	-	-	AB472710
D. sphenocarpa	TNS-F-21066	MAFF 241189	-	-	-	-	AB472726
D. sphenocarpa	TNS-F-21046	MAFF 241173	-	-	-	-	AB472708
Dacryoscyphus chrysochilus	KUN F45014	-	-	-	-	-	AY604567
Ditiola haasii	RoKi100	-	-	-	-	-	AF291314
D. radicata	UPS F-939957	-	-	-	-	-	MN595641
Femsjonia peziziformis	TNS-F-15737	MAFF 240152	-	-	-	-	AB299080
F. peziziformis	FO25100	-	-	-	-	-	AF291330
F. uniseptata	TNS-F-54018	-	-	-	-	-	LC222842
F. uniseptata	TNS-F-54019	MAFF 246082	-	-	-	-	LC222843
Guepiniopsis buccina	AFTOL-ID 888	-	-	-	-	-	AY745711
G. buccina	TNS-F-15738	MAFF 240153	-	-	-	-	AB299085
Heterotextus miltinus	PDD 107924	ICMP 21229	-	-	-	-	LC131402
H. miltinus	PDD 104962	ICMP 20435	-	-	-	-	LC131401
Unilacryma bispora	UPS F-941254	-	-	-	-	-	MN595670
U. bispora	UPS F-941274	-	-	-	-	-	MN595675
U. unispora	TNS-F-15731	MAFF 240146	-	-	-	-	AB299074
U. unispora	TNS-F-38904	-	-	-	-	-	AB712432
"Clade A" (12121-1B1, culture)	-	NBRC 110596	-	-	-	-	LC003886

"Clade A" (1401155-8, eDNA)	-	-	-	-	-	-	LC003988
"Clade B" (09161-1A3, culture)	-	NBRC 110590	-	-	-	-	LC003880
"Clade B" (1308156-2, eDNA)	-	-	-	-	-	-	LC003968
"Clade C" (HNo1115, fruiting body)	TNS-F-61322	-	-	-	-	-	LC003925
"Clade C" (1305153-8, eDNA)	-	-	-	-	-	-	LC003942
"Clade D" (HNo1088, fruiting body)	TNS-F-61298	NBRC 110568	-	-	-	-	LC003901
"Clade D" (HNo1111, fruiting body)	TNS-F-61318	-	-	-	-	-	LC003921
"Clade E" (HNo1102, fruiting body)	TNS-F-61311	-	-	-	-	-	LC003914
"Clade E" (HNo1104, fruiting body)	TNS-F-61312	-	-	-	-	-	LC003915
"Clade F" (03142-2A4, culture)	-	NBRC 110598	-	-	-	-	LC003890
"Clade F" (04111-2A2, culture)	-	NBRC 110599	-	-	-	-	LC003891
"Clade G" (11131-1C5, culture)	-	NBRC 110592	-	-	-	-	LC003884
"Clade H" (07152-2B1, culture)	-	NBRC 110589	-	-	-	-	LC003879
"Clade H" (09161-1C5, culture)	-	-	-	-	-	-	LC003881
"Clade I" (12121-1D8, culture)	-	NBRC 110597	-	-	-	-	LC003888
"Clade K" (10141-1F3, culture)	-	NBRC 110591	-	-	-	-	LC003883
"Clade K" (1307159-9, eDNA)	-	-	-	-	-	-	LC003964
**Outgroup**							
Exidia uvapsassa	AFTOL 461	-	-	-	-	-	AY645056
Pseudohydnum gelatinosum	AFTOL 1875	-	-	-	-	-	DQ520094

[1]INPA, Instituto Nacional de Pesquisas da Amazônia (Brazil); MIN, University of Minnesota (U.S.A.); PDD, Fungal and Plant Disease Collection (New Zealand).; TNS, National Museum of Nature and Science (Japan); TUMH, Tottori University Mycological Herbarium (Japan); UPS, Uppsala University (Sweden).

[2]CBS, Fungal Biodiversity Centre of the Centraalbureau voor Schimmelcultures (the Netherlands); ICMP, International Collection of Micro-organisms from Plants (New Zealand); NBRC, Biological Resource Center; MAFF, Culture collection of National Institute of Agrobiological Science; TUFC, Fungal culture collection of Tottori University (Japan); others, Forest Products Laboratory (USA).

[3]Sequences of eDNA are the representative sequences of each OTU.

[4]Cultures stopped the growth during preservation.

Supplementary Table 2 All results of AICc-based model selection for the effects of environmental factors on OTU richness detected according to fruiting-body collection

Model	(Intercept)	Decomposition stage	Forest age	Season	Year	df	logLik	AICc	delta	weight
5	0.054067221	-	-	+	-	4	-75.92144126	160.4398975	0	0.454798736
7	0.334174822	-	-0.004904093	+	-	5	-75.82954707	162.568185	2.128287594	0.156916087
6	0.14560586	-0.030617027	-	+	-	5	-75.90613395	162.7213588	2.281461348	0.145347047
13	0.30538165	-	-	+	+	6	-74.96005731	163.2124223	2.772524849	0.113703315
8	0.42571346	-0.030617027	-0.004904093	+	-	6	-75.81423975	164.9207872	4.480889741	0.048395659
15	0.58548925	-	-0.004904093	+	+	7	-74.86816311	165.4863262	5.046428767	0.036475493
14	0.396920289	-0.030617027	-	+	+	7	-74.94474999	165.6395	5.199602521	0.033786243
16	0.677027889	-0.030617027	-0.004904093	+	+	8	-74.85285579	167.9914259	7.551528417	0.010423791
1	-0.384845821	-	-	-	-	1	-88.06520249	178.1875478	17.74765038	6.36746E-05
3	-0.104738221	-	-0.004904093	-	-	2	-87.97330829	180.1205296	19.68063218	2.42228E-05
2	-0.293307182	-0.030617027	-	-	-	2	-88.04989517	180.2737034	19.83380593	2.24369E-05
9	-0.133531393	-	-	-	+	3	-87.10381853	180.5605782	20.12068079	1.94388E-05
4	-0.013199582	-0.030617027	-0.004904093	-	-	3	-87.95800098	182.2689431	21.82904568	8.27375E-06
11	0.146576208	-	-0.004904093	-	+	4	-87.01192434	182.6208636	22.18096614	6.93879E-06
10	-0.041992754	-0.030617027	-	-	+	4	-87.08851121	182.7740374	22.3341399	6.42721E-06
12	0.238114847	-0.030617027	-0.004904093	-	+	5	-86.99661702	184.9023249	24.46242749	2.21754E-06

Hyphen, Explanatory variable was not selected.

Supplementary Table 3 All results of AICc-based model selection for the effects of environmental factors on OTU richness detected according to culture isolation

Model	(Intercept)	Decomposition stage	Forest age	Season	Year	df	logLik	AICc	delta	weight
3	0.364868816	-	-0.023511467	-	-	2	-57.15718872	118.4882905	0	0.18310923
1	-0.944461609	-	-	-	-	1	-58.31612606	118.689395	0.201104499	0.165592609
4	-0.651882827	0.327131113	-0.023511467	-	-	3	-56.18443452	118.7218102	0.233519741	0.16293038
2	-1.961213226	0.327131109	-	-	-	2	-57.34337186	118.8606568	0.372366294	0.152002918
7	0.02839658	-	-0.023511467	+	-	5	-55.03792555	120.984942	2.496651531	0.05254958
5	-1.280933845	-	-	+	-	4	-56.19686289	120.9907407	2.502450233	0.052397441
6	-2.297685489	0.327131113	-	+	-	5	-55.2241087	121.3573083	2.869017825	0.043622538
8	-0.988355064	0.327131113	-0.023511467	+	-	6	-54.06517135	121.4226504	2.934359922	0.042220374
11	0.077186744	-	-0.023511467	-	+	4	-56.67370094	121.9444168	3.456126338	0.032525347
9	-1.232143681	-	-	-	+	3	-57.83263829	122.0182177	3.529927275	0.031347021
12	-0.9395649	0.327131113	-0.023511467	-	+	5	-55.70094675	122.3109844	3.82269393	0.027078387
10	-2.248895325	0.327131113	-	-	+	4	-56.85988409	122.3167831	3.828492632	0.026999991
13	-1.568615918	-	-	+	+	6	-55.71337512	124.7190579	6.230767456	0.008122997
15	-0.259285493	-	-0.023511467	+	+	7	-54.55443778	124.8588756	6.370585078	0.007574523
14	-2.585367561	0.327131113	-	+	+	7	-54.74062092	125.2312418	6.742951372	0.006287774
16	-1.276037136	0.327131113	-0.023511467	+	+	8	-53.58168358	125.4490814	6.960790972	0.005638891

Hyphen, Explanatory variable was not selected.

Supplementary Table 4 All results of AICc-based model selection for the effects of environmental factors on OTU richness detected according to eDNA analysis

Model	(Intercept)	Decomposition stage	Forest age	Season	Year	df	logLik	AICc	delta	weight
1	1.73128155	-	-	-	-	1	-162.8938891	327.8457493	0	0.313679792
2	1.530449466	0.066446732	-	-	-	2	-162.2960946	328.7686599	0.922910598	0.197732953
3	1.883582399	-	-0.002666529	-	-	2	-162.6714414	329.5193534	1.673604112	0.135852622
4	1.682751705	0.066477935	-0.002668211	-	-	3	-162.0733663	330.5049415	2.659192248	0.082994685
5	1.663505134	-	-	+	-	4	-161.1054653	330.8169913	2.971242024	0.071005098
9	1.68948062	-	-	-	+	3	-162.7069898	331.7721886	3.926439346	0.04404241
6	1.46263462	0.066466157	-	+	-	5	-160.5074964	331.9380697	4.09232039	0.040536893
7	1.817719855	-	-0.002691838	+	-	5	-160.8789139	332.6809048	4.835155479	0.02796056
10	1.488757724	0.066417667	-	-	+	4	-162.109457	332.8249746	4.97922536	0.026017256
11	1.840283883	-	-0.002632077	-	+	4	-162.4903462	333.5867529	5.741003667	0.017776384
8	1.616849358	0.066498323	-0.002693554	+	-	6	-160.2806556	333.8738113	6.028062002	0.015399602
12	1.63956331	0.066450299	-0.002633857	-	+	5	-161.8925199	334.7081166	6.86236737	0.010147111
13	1.621555213	-	-	+	+	6	-160.9221	335.1567001	7.310950823	0.008108373
14	1.420796166	0.066436939	-	+	+	7	-160.3243941	336.4265661	8.580816789	0.004297203
15	1.774309377	-	-0.002657363	+	+	7	-160.7014225	337.1806228	9.334873553	0.002947437
16	1.573551126	0.066470674	-0.002659184	+	+	8	-160.1034132	338.529407	10.68365776	0.001501619

Hyphen, Explanatory variable was not selected.

Supplementary Table 5 All results of AICc-based model selection for the effects of environmental factors on MPD (SES) detected according to eDNA analysis

Model	(Intercept)	Decomposition stage	Forest age	Season	Year	df	logLik	AICc	delta	weight
10	0.65465216	-0.18143529	-	-	+	5	-79.67987966	170.2828362	0	0.14066462
2	0.730718498	-0.18143529	-	-	-	3	-82.10654513	170.5712992	0.288462982	0.121771578
9	0.110346291	-	-	-	+	4	-81.08188454	170.7698297	0.486993444	0.11026447
1	0.186412629	-	-	-	-	2	-83.41760462	171.0116798	0.728843577	0.097705386
12	1.049722709	-0.18143529	-0.006870792	-	+	6	-79.20300034	171.7185007	1.435664433	0.068617408
14	0.791179088	-0.18143529	-	+	+	8	-76.80425337	171.9310874	1.648251134	0.061698086
4	1.111508201	-0.18143529	-0.006642769	-	-	4	-81.69042843	171.9869175	1.704081232	0.059999598
11	0.50541684	-	-0.006870792	-	+	5	-80.62359133	172.1702596	1.887423349	0.054743947
6	0.861404733	-0.18143529	-	+	-	6	-79.50475668	172.3220134	2.039177124	0.050743824
3	0.567202333	-	-0.006642769	-	-	3	-83.01666084	172.3915306	2.108694396	0.049010339
13	0.246873219	-	-	+	+	7	-78.32205755	172.4218929	2.139056629	0.048271926
5	0.317098864	-	-	+	-	5	-80.91355903	172.750195	2.467358731	0.040964224
16	1.202181963	-0.18143529	-0.00712441	+	+	9	-76.24800862	173.4468369	3.16400067	0.02891551
8	1.256924556	-0.18143529	-0.006878606	+	-	7	-79.02450963	173.826797	3.543960806	0.023912422
15	0.657876094	-	-0.00712441	+	+	8	-77.78926943	173.9011195	3.61828327	0.023040115
7	0.712618687	-	-0.006878606	+	-	6	-80.45211914	174.2167383	3.933902046	0.019676548

Hyphen, Explanatory variable was not selected.

Supplementary Table 6 All results of AICc-based model selection for the effects of environmental factors on MNTD (SES) detected according to eDNA analysis

Model	(Intercept)	Decomposition stage	Forest age	Season	Year	df	logLik	AICc	delta	weight
10	0.602398308	-0.288770079	-	-	+	5	-66.57647288	144.0760227	0	0.563796112
12	1.015941021	-0.288770079	-0.007192047	-	+	6	-65.81768255	144.9478651	0.871842435	0.364589282
14	0.730403716	-0.288770079	-	+	+	8	-65.80556593	149.9337125	5.857689824	0.030139837
16	1.148899478	-0.288770079	-0.007254293	+	+	9	-65.01683296	150.9844856	6.908462916	0.017822491
9	-0.26391193	-	-	-	+	4	-71.4638803	151.5338212	7.457798527	0.013541965
11	0.149630784	-	-0.007192047	-	+	5	-70.80359683	152.5302706	8.454247914	0.008228212
13	-0.135906522	-	-	+	+	7	-70.7930681	157.363914	13.2878913	0.000733995
2	0.461730891	-0.288770079	-	-	-	3	-76.03605899	158.4303269	14.35430425	0.00043065
15	0.282589241	-	-0.007254293	+	+	8	-70.10871795	158.5400165	14.46399387	0.000407667
4	0.842723921	-0.288770079	-0.006646316	-	-	4	-75.54126636	159.6885933	15.61257065	0.00022956
1	-0.404579346	-	-	-	-	2	-79.83902069	163.854512	19.7784893	2.85942E-05
6	0.582340378	-0.288770079	-	+	-	6	-75.48460393	164.2817079	20.20568518	2.30948E-05
3	-0.023586316	-	-0.006646316	-	-	3	-79.39480856	165.1478261	21.0718034	1.49775E-05
8	0.966570769	-0.288770079	-0.006682268	+	-	7	-74.97684137	165.7314605	21.65543785	1.11867E-05
5	-0.283969859	-	-	+	-	5	-79.34397896	169.6110348	25.53501217	1.60792E-06
7	0.100260532	-	-0.006682268	+	-	6	-78.88885977	171.0902195	27.01419686	7.67474E-07

Hyphen, Explanatory variable was not selected.
